# Ligand and Structure-Based Approaches for the Identification of Peptide Deformylase Inhibitors as Antibacterial Drugs

**DOI:** 10.3390/ijms17071141

**Published:** 2016-07-15

**Authors:** Jian Gao, Li Liang, Yasheng Zhu, Shengzhi Qiu, Tao Wang, Ling Zhang

**Affiliations:** 1 Jiangsu Key Laboratory of New Drug Research and Clinical Pharmacy, Xuzhou Medical University, Xuzhou 221004, Jiangsu, China; 18852143485@163.com (L.L.); 18151865223@163.com (Y.Z.); neoevens@hotmail.com (S.Q.); lswangtao@163.com (T.W.); zhamgling1999@163.com (L.Z.); 2 Jiangsu Center for the Collaboration and Innovation of Cancer Biotherapy, Xuzhou Medical University, Xuzhou 221004, Jiangsu, China

**Keywords:** peptide deformylase, pharmacophore model, high-throughput virtual screening, molecular docking, antibacterial drug

## Abstract

Peptide deformylase (PDF) is a metalloprotease catalyzing the removal of a formyl group from newly synthesized proteins, which makes it an important antibacterial drug target. Given the importance of PDF inhibitors like actinonin in antibacterial drug discovery, several reported potent PDF inhibitors were used to develop pharmacophore models using the Galahad module of Sybyl 7.1 software. Generated pharmacophore models were composed of two donor atom centers, four acceptor atom centers and two hydrophobic groups. Model-1 was screened against the Zinc database and several compounds were retrieved as hits. Compounds with Qfit values of more than 60 were employed to perform a molecular docking study with the receptor *Escherichia coli* PDF, then compounds with docking score values of more than 6 were used to predict the in silico pharmacokinetic and toxicity risk via OSIRIS property explorer. Two known PDF inhibitors were also used to perform a molecular docking study with *E. coli* PDF as reference molecules. The results of the molecular docking study were validated by reproducing the crystal structure of actinonin. Molecular docking and in silico pharmacokinetic and toxicity prediction studies suggested that ZINC08740166 has a relatively high docking score of 7.44 and a drug score of 0.78.

## 1. Introduction

In this day and age, in spite of the rapid development of antibacterial drugs [1,2,3], infectious diseases remain the second-leading cause of death worldwide [4,5]. In addition, many of these agents share the same targets, and the actual number of antibacterial targets is very limited [6], more and more bacteria also have developed resistance to medicines used for treatment of human infections [4,7]. Therefore, it is a major concern that we should search for new antibacterial agents with a high potency against drug-resistant pathogens. Searching for good and novel targets is vital. Theoretically, the target should be part of an essential pathway in the pathogens and be absent in human cells [4]. The recent research has confirmed that peptide deformylase (PDF) is a possible target to develop new antibacterial drugs [8,9]. PDF is an essential bacterial metalloenzyme, which deformylates the *N*-formylmethionine of newly synthesized polypeptides [10,11]. Recent studies also demonstrated that PDF also can be found in most eukaryotes, including parasites, plants, and mammals [12,13], but human PDF is overexpressed in cancer cells and not in normal cells [14]. Hence, PDF inhibitors exhibit little effect over normal human cells, which make them an appealing target for the design of novel antibacterial agents [15]. 

**Figure 1:**
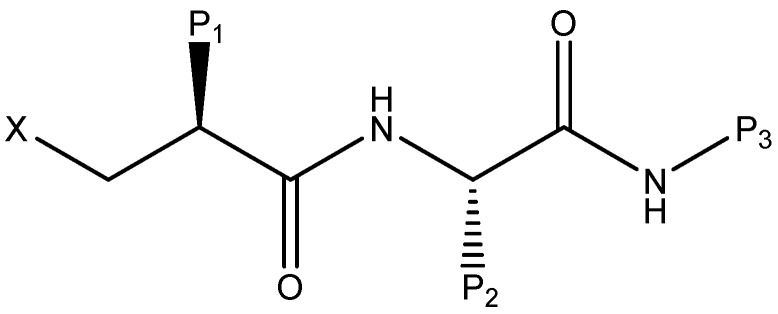
Proposed general structure for peptide deformylase (PDF) inhibitor.

Naturally occurring antibiotics, like actinonin, inhibit the activity of the PDF enzyme [16,17]. Therefore, it is feasible to synthesize a new type of inhibitors by the framework of the crystal structure of PDF [18]. On account of the study of actinonin, many PDF inhibitors with antibacterial activity have been synthesized [19,20,21]. In these structures of inhibitors (Figure 1), X represents a metal chelator, which is usually hydroxamic acid or *N*-formyl-*N*-hydroxylamine, considered to be essential to the antibacterial activity of these inhibitors. Positions P_1_ and P_2_ of the inhibitor are usually hydrophobic side chains, providing additional binding energy for PDF inhibition. The P_3_ position, however, is usually amenable to different substitutions.

Computer-aided drug design (CADD) is presently a key component in the process of drug discovery and development, as it offers great promise to drastically reduce cost and time requirements [22]. However, to our knowledge, there are very few reports on the pharmacophore study of PDF inhibitors. The aim of this study is to obtain a pharmacophore model based on inhibitors containing hydroxamic acid or *N*-formyl-*N*-hydroxylamine that could provide a rational hypothetical picture of the chemical features responsible for the antibacterial activity of these compounds. Therefore, PDF inhibitors with high antibacterial activity were used to establish pharmacophore models with the Galahad module which is a convenient method for finding a pharmacophore and aligning ligand molecules that bind at a common target site in Sybyl 7.1 [23]. Based on this pharmacophore knowledge, high-throughput virtual screening of databases and molecular docking studies can be used to develop new and potentially more active compounds for the treatment of infectious diseases [24,25]. Therefore, with the increasing of multidrug-resistant strains, ligand and structure-based antibacterial drug design may contribute to discovering novel antibacterial drugs [26,27].

## 2. Results and Discussion

### 2.1. Results of Pharmacophore Mapping

**Figure 2:**
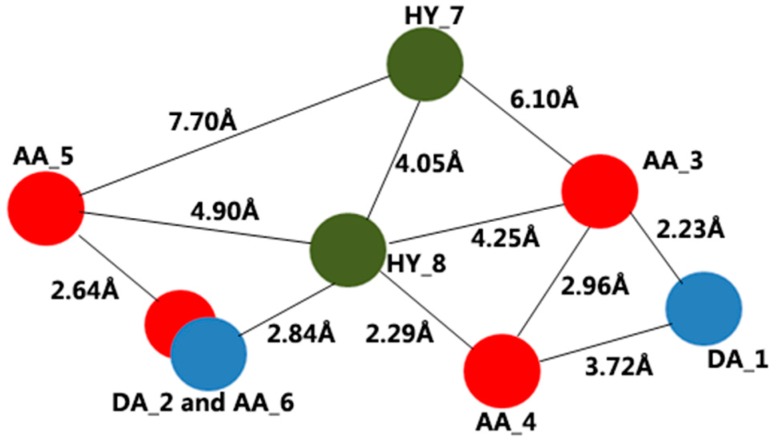
Obtained pharmacophore model consisting of two donor atom centers (DA), four acceptor atom centers (AA) and two hydrophobic groups (HY).

With the purpose of building pharmacophore models, we chose eighteen potent compounds. Based on the Galahad module in Sybyl 7.1, two models were obtained. The two models are equal with the same specificity value of 3.65. Specificity is a logarithmic indicator of the expected discrimination for each query, based on the number of features it contains and the degree to which the features are separated in space. The result of the pharmacophore hypothesis is shown in Table 1. In addition, the pharmacophore model is shown in Figure 2. The best model contains two donor atom centers, four acceptor atom centers and two hydrophobic groups.

### 2.2. Virtual Screening Analysis

The best pharmacophore model was employed as a 3D search query against the Zinc database. A great deal of molecules was obtained from the Zinc database after virtual screening. Then, the model was performed as a Flex search query against these molecules. For the molecular docking study, 43 molecules were selected with a Qfit value of more than 60. The Qfit value represents how closely the ligand atoms matched with the 3D search query. ZINC08740166, with the highest Qfit value, matches the pharmacophore model: the oxygen atoms of the carbonyl group of ZINC08740166, which accepts a proton, are located at AA_3 and AA_4, and the NH group of pyrrolidine, which provides a proton, is located at DA_2.

### 2.3. Validation of Molecular Docking

**Figure 3:**
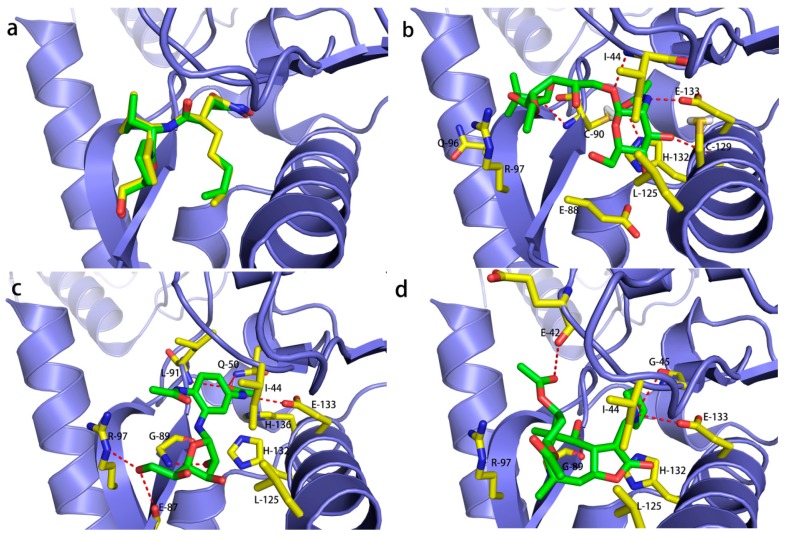
(**a**) Superposition of the crystal structure of *Escherichia coli* PDF-actinonin (colored in green) and its docked conformation (colored in yellow). The binding mode of three compounds in the *E. coli* PDF binding pocket: (**b**) ZINC12660672; (**c**) ZINC12652500 and (**d**) ZINC08740166. The red color bonds indicate the hydrogen bonds between compound and amino acids (colored by yellow).

With the aim of testing and validating molecular docking, molecular docking studies were applied to compare the conformations from the docked structure and the crystal structure (Figure 3a). The superposition of the crystal structure of *Escherichia coli* PDF (PDB:1G2A) complexed with actinonin and the docked conformation further demonstrates the accuracy of the molecular docking results, which indicates that molecular docking studies for other compounds are also feasible.

### 2.4. Molecular Docking Analysis

**Figure 4:**
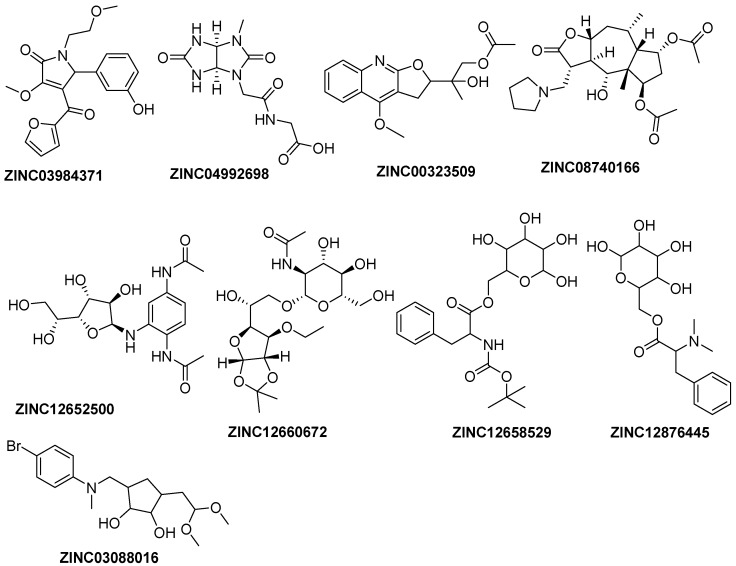
Structures of retrieved hits from the Zinc database with docking score values of more than 6.

To further explore the potential binding modes between *E. coli* PDF and identified hits, we performed a molecular docking study, in order to identify novel compounds with a good antibacterial activity. Eight compounds with total score values of more than 6 (Figure 4) and two known potent PDF inhibitors, A and J, were docked in *E. coli* PDF. Compounds A and J used to perform docking studies are from a crystal structure that suggested the availability of docking results and the docking score values of compound A and J are also relatively high, which indicate they have strong interaction with *E. coli* PDF. The results of molecular docking are shown in Table 2. 

### 2.5. Binding Mode of ZINC12660672, ZINC12652500 and ZINC08740166 in E. coli PDF

The overall binding of ZINC12660672, ZINC12652500 and ZINC08740166 in *E. coli* PDF is illustrated in Figure 3b–d. Molecular docking studies suggest that these compounds have a similar binding mode in the active site. Although these compounds are very different in structure, they are able to fit in the same binding pocket. The compounds have showed hydrogen bond interactions with several residues such as Ile44, His132 and Glu133.

Molecular docking studies showed that ZINC12660672 (Figure 3b) has the highest docking score (8.10) among the three compounds, forming five hydrogen bonds with the active site residues of PDF. The oxygen atom of the hydroxyl group formed a hydrogen bond with the NH group on the imidazole ring of His132. The NH group attached to the tetrahydropyrane group formed a hydrogen bond with the oxygen atom of the carboxyl group of the side chain of Glu133. Another three hydrogen bonds established a network with the backbones of Cys90, Cys129 and Ile44. In addition, there was a hydrophobic effect with the side chains of Gln96, Arg97, Glu88, Leu125 and Ile44.

ZINC12652500, compared with ZINC12660672, has a close docking score of 8.05, and formed six hydrogen bonds with active site residues of PDF (Figure 3c). The NH group connected with the phenyl ring showed a hydrogen bond interaction with the oxygen atom of the carboxyl group of the side chain of Glu133. The oxygen atom of the 3-hydroxyl group of tetrahydrofuran showed a hydrogen bond interaction with the NH group of the backbone of Gly89. The hydroxyl group at the terminal of ZINC12652500 showed hydrogen bond interactions with both the NH group of the side chain of Arg97 and the oxygen atom of the carboxyl group of Glu89. Another two hydrogen bonds established a network with the backbone of Leu91 and the side chain of Gln50. Additionally, ZINC12652500 showed hydrophobic interactions with several residues, which formed a hydrophobic pocket including Ile44, Leu125, His132 and His136.

ZINC08740166 has a docking score of 7.44 and showed hydrogen bond interactions with Gly89, Glu133, Gly45 and Glu42 (Figure 3d). The NH group of pyrrolidine also showed a hydrogen bond interaction with the oxygen atom of the carboxyl group of Glu133 and Gly45. Additionally, two hydrogen bonds were formed with the backbones of Gly89 and Glu42. Within the cavity of the active site, His132, Arg97, Ile44 and Leu125 probably generate a hydrophobic effect. Interestingly, these three compounds are very different in structure with the previously reported actinonin, but they show identical hydrogen bond interactions with several residues, such as Ile44, His132 and Glu133.

### 2.6. In Silico Pharmacokinetic and Toxicity Prediction

Three compounds, ZINC12660672, ZINC12652500 and ZINC08740166, with high docking scores were selected to perform in silico pharmacokinetic and toxicity prediction studies. Mutagenicity, tumorigenicity, irritant and reproductive effects of these compounds were predicted and the results showed that they appear to pose no risk of toxicity (Table 3). Furthermore, we performed docking studies using these three compounds with human PDF, and the docking score values were less than 6, which further illustrates that these three compounds are likely to have little adverse effects on the human body. The value of drug score, drug-likeness and solubility were also predicted by pharmacokinetic prediction (Table 3). The value of the drug score is used to judge the compound’s overall potential to qualify as a drug candidate. Compared with ZINC12660672 and ZINC12652500, ZINC08740166 has a relatively high drug score of 0.78 and drug-likeness of 4.62, which suggests that ZINC08740166 may be a potential antibacterial drug.

## 3. Materials and Methods

### 3.1. Database Search and Calculation Method

Based on previous studies [4], a series of PDF inhibitors were obtained from the Research Collaboratory for Structural Bioinformatics (RCSB) Protein Data Bank (PDB) and used for the generation of pharmacophore models. The generation of pharmacophore models, high-throughput virtual screening and molecular docking studies were performed in Sybyl 7.1. Retrieved hits for docking studies were added to the hydrogen atoms and the charge is given to the Gasteiger-Huckel, which optimizes the structure of compounds and contributes to the combination between compounds and protein. Energy minimizations were performed using the Tripos force field with an energy optimization gradient convergence criterion of 0.001 kcal/mol·Å and a maximum optimal step size of 1000.

### 3.2. Generation of Pharmacophore Model

**Figure 5:**
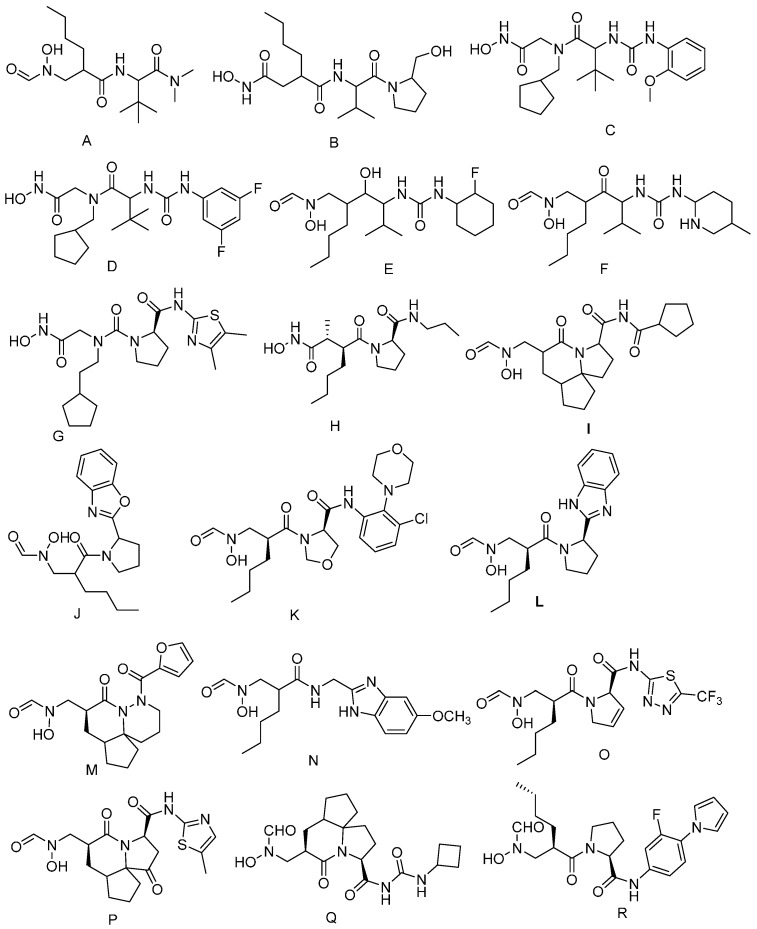
Chemical structures of known PDF inhibitors used for the generation of pharmacophore models.

The compounds (Figure 5) selected were used to establish pharmacophore models with the Galahad module. The parameters were set as follows: The population size, max generation, and mols required to hit showed values of 2, 5, and 4, respectively. Then, two identical models were generated. The obtained models included hydrophobic, hydrogen bond acceptor and hydrogen bond donor features. The compounds used to build the pharmacophore model had been hit and the most active compound in each class training set showed a good fit with all features of the pharmacophore proposed.

### 3.3. High-Throughput Virtual Screening

High-throughput virtual screening emerged as an important tool in our quest to access novel drug-like compounds [28], which has been widely applied in early-stage drug discovery [29]. After the generation of the pharmacophore models, the best model was used to perform a 3D search query from the Zinc database to obtain hit molecules. Then, the best model, as a Flex search query, was screened again against the hit molecules for retrieving the potent hit molecules. 

### 3.4. Molecular Docking

The Surflex-Dock module of Sybyl 7.1 software was used for molecular docking studies to predict ligand-receptor interaction modes, and for hit identification by structure-based virtual screening [30]. This program allows ligand structures to dock in a conformationally flexible manner to a protein and adopts a rigid-body protein approximation to speed up the calculation of binding free energy. Forty-three retrieved hits with Qfit values of more than 60, along with two known PDF inhibitors, were employed to perform the molecular docking study with *E. coli* PDF. Dock uses an empirical scoring function and a patented search engine to dock ligands into a protein’s binding site [31]. Blank ProtoMol generation was applied for the determination of the active site of the *E. coli* PDF. Water molecules were removed, and hydrogen atoms were added during protein preparation.

### 3.5. Validation of Molecular Docking

Although molecular docking further elucidates the binding mode of compounds, there are still some deficiencies due to the fact that the receptor was regarded as a rigid structure while performing the docking study. Therefore, it is essential to validate the accuracy of these docking methods. In view of this, molecular docking studies were also applied to the crystal structure of *E. coli* PDF-actinonin with the same docking parameters. Then, we compared the molecular conformations from the docking studies with the one from the crystal structure using PyMOL software (Schrödinger, New York, NY, USA) [32].

### 3.6. In Silico Pharmacokinetics and Toxicity Studies

In silico pharmacokinetics and toxicity were predicted using OSIRIS property explorer, which uses Chou and Jurs algorithm, based on computed atom contributions [33].

## 4. Conclusions

Ligand and structure-based approaches, including pharmacophore-based virtual screening, molecular docking and in silico pharmacokinetic and toxicity prediction studies were used to search for novel potent PDF inhibitors. The generated model-1 was employed to screen the Zinc database. Then we performed a molecular docking study using compounds with Qfit values of more than 60, after which two known PDF inhibitors were chosen as reference molecules. Based on docking score values of more than 6, we selected three compounds to perform pharmacokinetic predictions. Considering the predicted results in in silico pharmacokinetic and toxicity prediction studies, including values of drug score, drug-likeness and solubility, ZINC08740166 represents a valuable antibacterial candidate drug for the treatment of infectious disease. These findings provide insight to elucidate the binding pattern of PDF inhibitors and to help in the rational structure-based design of novel PDF inhibitors with improved potency.

## Figures and Tables

**Table 1:** Results of the pharmacophore hypothesis generated using the Galahad module.

Model	Specificity	*N*-hits ^a^	Feats ^b^	Energy ^c^	Sterics ^d^	Hbond ^e^	Mol-Qry ^f^
1	3.65	18	8	9.03	690	181.40	48.51
2	3.65	18	8	9.03	690	181.40	48.51

^a^ *N*-hits: actual number hit; ^b^ Feats: total number of features in the model query; ^c^ Energy: total energy of the model; ^d^ Sterics: steric overlap for the model; ^e^ Hbond: pharmacophoric concordance; ^f^ Mol-Qry: agreement between the query tuplet and the pharmacophoric tuplet for the ligands as a group.

**Table 2:** Docking score values of the retrieved hits and known PDF inhibitors (A and J).

Compound	Total Score ^a^	Crash ^b^	Polar ^c^
J	9.41	−2.60	5.75
ZINC12660672	8.10	−2.14	1.29
ZINC12652500	8.05	−2.58	6.25
ZINC08740166	7.44	−2.94	1.67
A	7.11	−2.31	4.56
ZINC03984371	6.51	−1.79	1.22
ZINC04992698	6.40	−0.54	6.68
ZINC12658529	6.21	−2.71	3.32
ZINC03088016	6.18	−0.96	5.26
ZINC00323509	6.12	−1.18	2.11

^a^ Total Score: total docking score; ^b^ Crash: degree of inappropriate penetration by the ligand into the protein and of interpenetration between ligand atoms that are separated by rotatable bonds; ^c^ Polar: contribution of the polar non-hydrogen bonding interactions to the total score.

**Table 3:** In silico pharmacokinetic and toxicity prediction study of ZINC12660672, ZINC08740166 and ZINC12876445.

Parameters	ZINC12660672	ZINC08740166	ZINC12652500
Mutagenicity ^a^	NO	NO	NO
Tumorigenicity ^b^	NO	NO	NO
Irritant ^c^	NO	NO	NO
Reproductive effect ^d^	NO	NO	NO
Solubility ^e^	−1.37	−3.01	−1.93
cLogP ^f^	−2.24	1.26	−1.61
Drug-likeness ^g^	−6.68	4.62	−1.86
Molecular weight	451.0	437.0	369.0
Drug score ^h^	0.41	0.78	0.15

^a^ Mutagenicity: induction of permanent transmissible changes in the structure of the genetic material of cells or organisms; ^b^ Tumorigenicity: process by which neoplastic cells grown in tissue culture form tumors; ^c^ Irritant: stimulus from the compound which causes irritation; ^d^ Reproductive effect: adverse effect of compounds that interferes with the reproductive organs of an organism, such as genitalia and gonads; ^e^ Solubility: estimated logS value, a unit stripped logarithm (base 10) of the solubility measured in mol/L; ^f^ cLogP: logarithm of the partition coefficient of the compound between n-octanol and water log (octanol/water); ^g^ Drug-likeness value of compound’s drug-like properties; ^h^ Drug score: combines drug-like properties, cLogP, logS, molecular weight and factors of toxicity risk management in one handy value used to judge the compound’s overall potential to qualify as a drug candidate.
